# Nerve electrical stimulation enhances osseointegration of implants in the beagle

**DOI:** 10.1038/s41598-019-41471-z

**Published:** 2019-03-20

**Authors:** Ping Zhou, Fei He, Bin Liu, Shicheng Wei

**Affiliations:** 1 School of Stomatology, Lanzhou University, Lanzhou, Gansu PR China; 2 Center for Biomedical Materials and Tissue Engineering, Academy for Advanced Interdisciplinary Studies, Peking University, Beijing, PR China; 3 Central Laboratory, Peking University School and Hospital of Stomatology, National Engineering Laboratory for Digital and Material Technology of Stomatology, Beijing Key Laboratory of Digital Stomatology, Beijing, PR China

## Abstract

Dental implantation has been the primary method for the treatment of tooth loss, but longer than 3 months healing times are generally required. Because immediate load implants are suitable only for certain categories of implant patients, it has value to develop a novel method to facilitate the implant-bone osseointegration process. Cylindrical titanium implants were implanted in the tooth sockets of beagles, and microelectrode stimulation of the sympathetic nerves in the infraorbital nerve was performed after implantation for 1 week. The authors found that one-sided nerve stimulation was shown to evoke consistent electric potential changes in both sides of the infraorbital nerves. Moreover, after 4 weeks of implantation, more new bone was clearly observed around the implants in the beagles that received electrical stimulation treatment than was observed in the control animals. Furthermore, a higher mineralization density was measured in the new peri-implant bone tissues of the stimulated beagles when compared to controls. These results demonstrate that the simple and safe physical method of microelectrode stimulation to sympathetic nerves can promote the formation of new bone and the osseointegration of implants. This technique is worth promoting and has the potential to reduce the healing time of dental implantation in future clinical cases.

## Introduction

Tooth loss leads to problems with chewing, pronunciation, appearance, and mental health. The widely used technology of dental implantation is considered to be the best method to repair missing teeth^1^. Once implants are placed into the bone tissue, multiple reactions, such as protein absorption, osteoblast adhesion, bone formation, and bone reconstruction, occur serially at the interface between the implant and the bone. The end result of these reactions is a stable condition called osseointegration^2^. Good bone-implant osseointegration is considered to be the foundation of successful dental implantations. Therefore, many researchers and dentists have made substantial efforts to promote the osseointegration of implants, which is a strong focus of the ongoing research in dental implantation.

Until recently, other than improvements to the surgical process, surface modification technologies have been the main approach to accelerate the osseointegration of implants. The surface modification of implants promotes the formation of new bone, increases the stability of the primary implant, and improves the performance of implant-bone osseointegration^3–7^. However, in clinical cases, a healing time that exceeds 3 months is required for the osseointegration of implants. Additionally, crown restoration and occlusal force are generally unacceptable during the implant-bone osseointegration process. Many patients forego dental implants due to the excessive timeframe and the discomfort of the process. Therefore, the authors believe that efforts should be made to shorten the healing time of dental implants, which could dramatically promote the application of dental implantation^8^. Recently, a method of immediate implants and immediate loading was developed to solve the problem of delayed restoration. However, it is only suitable for certain categories of implant patients, and there are a number of clinical cases in which immediate loading cannot be performed without a serious risk of failure. Additionally, because of the adverse effects on implant-bone osseointegration and alveolar bone crest maintenance, the failure rate of immediate-loading implants is significantly higher than that of delayed-loading implants^9–11^. As a result, the authors wanted to develop an approach to accelerate the processes of new bone formation and osseointegration to reduce the time interval between dental implantation and crown restoration.

It has been established that the adhesion and differentiation of osteoblasts are the most important processes in implant-bone osteointergration^2^. The cellular behaviour of osteoblasts is regulated by both numerous growth factors, including transforming growth factor beta, fibroblast growth factor, insulin-like growth factors, and bone morphogenetic protein, and by the nerves and neurohumor^12^. Unfortunately, most investigations of dental implants, including the previously mentioned surface modification technologies, focus on growth factors and their related signaling pathways, while few studies consider the regulation of nerves and neurohumor. Sympathetic nerves are widely distributed in bone tissue and play an important role in the regulation of bone formation via a number of adrenergic receptors in osteoblasts^12^. For example, hypothalamic leptin can react with the sympathetic nervous system, thereby regulating bone formation^13–15^. Many researchers have successfully regulated the bone formation process by using drugs or by transecting the sympathetic nerve of the bone, though the mechanisms of action for these methods of regulation are not clearly understood^16–18^. These studies showed that sympathetic nervous regulation has great potential to promote the formation of implant-bone osseointegration.

In the present study, the sympathetic nerves of the infraorbital nerve in beagles were stimulated with microelectrodes to induce changes in their electrical signal, and the effects of the electrical stimulation on new bone formation and implant-bone osseointegration were investigated. The authors believe that our simple and safe method of microelectrode stimulation holds great promise to accelerate the implant-bone osseointegration process in clinical cases.

## Materials and Methods

### *In vivo* surgery

Surgical implantations were conducted on eight female beagles that aged 2–3 years and weighing approximately 10 kg. Pure titanium were purchased from Northwest Institute For Non-ferrous Metal Research (Xi’an, China), and processed into cylindrical implants with diameter of 4.0 mm and length of 7.0 mm. Prior to the *in vitro* testing, the implants were sterilized by gamma radiation using a total dose of 25 KGγ. The beagles were anaesthetized using an intravenous injection of 1% pentobarbital at 80 mg·kg^−1^. Then, all maxillary lateral incisors of each of the eight beagles were extracted and 16 implants were immediately placed into each of the extraction sockets. All implants were covered by mucoperiosteal flaps and closed for submerged healing. Unfortunately, two failed implant in two animals were found by X-ray after 1 week of surgery, and these two implants were removed using the previously described method for tooth extraction. Eight beagles were divided into group A and group B, and each group was randomly assigned one-single implant beagle and three double-implant beagles. Fluorochrome of tetracycline (10 mg·kg^−1^ of body weight) and calecein (15 mg·kg^−1^ of body weight) obtained from Sigma-Aldrich (USA) were administered to analyze the osteogenic activity at 2 and 4 weeks. The animals were sacrificed after implantation for 4 weeks by an intracardiac injection of 10% potassium chloride (0.5 mL·kg^−1^). The maxillae of killed animals were obtained and promptly fixed in 10% formalin, then dehydrated in a graded series of alcohols. In the present study, all procedures for animal experiments were approved by the Institutional Animal Care Committee of Peking University (license number:AAIS-WeiSC-4), and the experiments were performed in accordance with the approved guidelines and regulations.

### Neural microelectrodes stimulation

Neural microelectrodes were applied to monitor the electric potential of sympathetic nerve fibers in the infraorbital nerves of beagles as previously reported^19–21^. Under general anaesthesia as described above, the skin above both suborbital foramens of each group B animal were bilaterally removed. The authors then dissected upwards towards the infraorbital nerves. A pair of metal microelectrodes were inserted bilaterally into the infraorbital nerves and reference electrodes were inserted into the soft tissue surrounding the infraorbital nerves. The electric potential of the sympathetic nerve fibers in the infraorbital nerve was detected in real time using a multiple physiologic recorder (Landy0602; Beijing Jing Shida technology development co., LTD, China).

As shown in figure S1, blood oxygen saturation (SpO_2_) and heart rate (HR) were detected in real time using an ECG monitor for each beagle in group B. One week after implantation, at a location that exhibited the most evident carotid pulse (near the thyroid cartilage), unanaesthetized group B animals were electrically stimulated transcutaneously using an electrical stimulator at 100 mv (voltage) and 1.5 Hz (frequency) for 45 min each day for three weeks. These setting parameters were chosen according to the SpO_2_ and heart rate results. Throughout the four-week study, no electrical stimulation was performed on the group A animals.

### Microcomputed tomography analysis

Serial micro-CT images of the implantation sites were obtained using a SkyScan 1076 scanner (Bruker, Germany) at 100 kV (X-ray source voltage), 80 µA (beam current), 900 msec (exposure time), 9 µm (resolution), 0.4° (rotation step), and 180° (rotation angle). The three-dimensional regenerated bone was reconstructed from micro-CT images using the CTAN software package (Skyscan). For the bone surrounding the implants at a distance between 0.25 mm and 2.5 mm, parameters such as percent bone volume (BV/TV), trabecular thickness (Tb.Th), trabecular number (Tb.N) and trabecular separation (Tb.Sp) were measured. Each of the 14 implants were tested in triplicate.

### Histological analysis

Dehydrated bone samples with implants were embedded in methyl methacrylate resin by a microtome (SP1600; Leica, Wetzlar, Germany), and cut into 30-µm-thick sections. Four sections were prepared for each of the 14 implant samples. An IX71 inverted fluorescence microscope (Olympus, Japan) was applied to observe the ingrowth of new bone and the integration of implants with the host tissue.

### Statistical analysis

All quantitative data are expressed as mean ± standard deviations. Statistical analysis was performed by one-way analysis of variance (ANOVA) followed by Tukey’s multiple comparison tests using SPSS 13.0. Statistical significance was accepted at p < 0.05.

## Results and Discussion

### Microelectrode stimulation of sympathetic nerves

To investigate the influence of microelectrode stimulation on bone healing with titanium implants, sixteen cylindrical titanium implants were implanted into each socket of an extracted maxillary anterior tooth in eight female adult beagles. After 1 week of implantation, the sympathetic nerves of the infraorbital nerves in 4 beagles with 7 implants (group B) were stimulated daily by microelectrodes for 3 weeks. The other 4 beagles with 7 implants (group A) underwent no treatment and served as controls. Anatomic studies show that the sympathetic nerves in the jaw arise from the superior cervical sympathetic ganglion^22^. Postganglionic fibers of the superior cervical sympathetic ganglion travel upward along the carotid artery and become a component of the trigeminal ganglion^19^. The sympathetic ganglion that runs down the outside of the skull accompanies the sensory fibers of the trigeminal ganglion, and both travel into the craniofacial tissues. To meet the criteria for clinical application, a location near the thyroid cartilage that presented the most apparent carotid pulse and contained the body surface projection area of the superior cervical sympathetic ganglion, was chosen for the transcutaneous electrical stimulation using an electrical stimulator. It was reported that daily stimulation with a pulsed electromagnetic field for 8 h for 4 days could promote the proliferation, differentiation and extracellular matrix synthesis of MG63 cells^22^. To avoid this effect, transcutaneous electrical stimulation to the surface of the superior cervical sympathetic ganglion of beagles for 45 min was applied each day.

As shown in figure S2, the heart rate (HR) of group B beagles decreased slightly after microelectrode stimulation for 1 min, but the HR value gradually returned to normal after 3 min of treatment. The oxygen saturation (SpO_2_) results were similar result for group B animals during the 3 min of microelectrode irritation. These results demonstrated that the microelectrode stimulation of the sympathetic nerves at 100 mv (voltage) and 1.5 Hz (frequency) was safe for beagles. It is worth noting that beagles with similar physical signs were applied for surgical implantations and electrical stimulation in our study, which is the reason why there was no individual variation in HR and SpO2 to the same electrical stimulation. However, because the physical condition of each human body could be quite different, the effect of parameter settings of electrical stimulation on HR and SpO2 of beagles with various physical signs such as gender, age and weight should be investigated prior to human study in the future. After obtaining the safe value range of each parameters for the electrical stimulation of different beagles, further study will be conducted to analyses the effect of electrical stimulation to sympathetic nerves on the formation of new bone in implanted beagles. Finally, several group of parameter settings were applied for the electrical stimulation to beagles with various type of physical signs, aiming to ensure safety and achieve good performance in accelerating new bone formation in implanted beagles at the same time. The results of these experiments will exhibit great value in promoting the application of microelectrode stimulation of sympathetic nerves to reduce the healing time of dental implantation in future clinical cases.

### Electric potential assay

**Figure 1:**
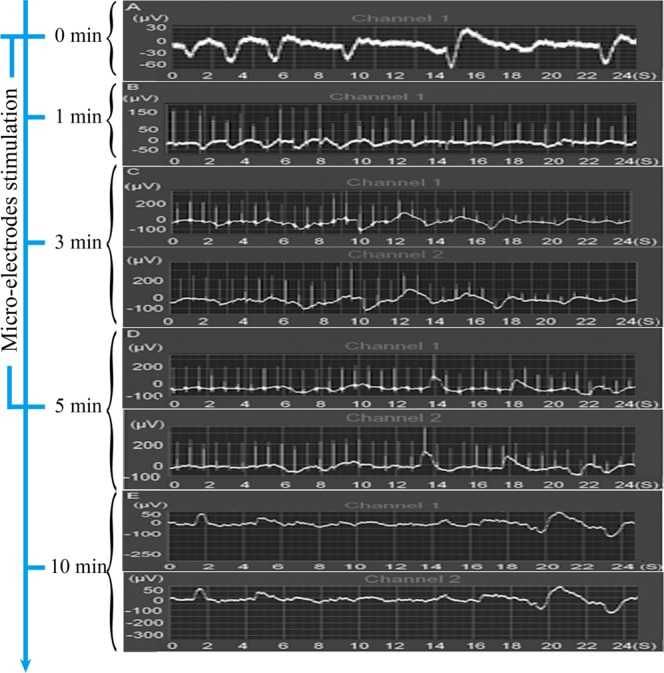
The electric potential of sympathetic nerve fibers in the infraorbital nerve of beagles before (**A**) and after microelectrode stimulation for various times (1 min, 3 min and 5 min) (**B–D**) was measured in real time by a multiple physiologic recorder. The stimulation was stopped for 5 min, and the electric potential was detected again. (**E**) In this figure, channel 1 and channel 2 were applied to detect the electric potential of the left and right sympathetic nerve fibers, respectively.

The curve of electric potential change versus time (s) for the sympathetic nerve fibers in the infraorbital nerve of beagles was detected in real time using a multiple physiologic recorder (Fig. 1). The voltage of the sympathetic nerve fibers at a resting state was approximately −30 μV to −60 μV, with a frequency of 0.3 Hz to 0.4 Hz (Fig. 1A). Although changes were found in the curve of the electric potential change versus time (s), the voltage of the sympathetic nerve fibers in beagles remained negative and maintained similar frequencies to the resting state after 1 min of stimulation (Fig. 1B). The voltage values of the positive and negative electric potentials both increased to approximately 100 μV after 3 min of stimulation (Fig. 1C), indicating that the microelectrode stimulation successfully changed the electric potential of the sympathetic nerve fibers in the infraorbital nerve of beagles at this time point. Upon further stimulation for 2 min, a few peaks of the positive electric potential were found in the curve of the electric potential change versus time (s) (Fig. 1D). Finally, the maximum voltage value of the positive electric potential decreased to approximately 50 μV when the stimulation was stopped for 5 min, which suggests that the impact of microelectrode stimulation on the electric potential of the sympathetic nerve fibers was transient (Fig. 1E). Additionally, as shown in Fig. 1C–E, the electric potential changes were found in both the left and right infraorbital nerves of the beagles, although electrical stimulation was only conducted on the surface of the right superior cervical sympathetic ganglion. Moreover, the electric potential change versus time (s) curves of channel 1 (left) and channel 2 (right) were highly consistent (Fig. 1), indicating that the implants that were implanted in the left or right side of the beagles received the same electric stimulation.

Classical neurotransmitters and co-transmission are the two kinds of neurotransmitter release patterns in neuronal cells, both of which are regulated by the frequency of electrical stimulation^23,24^. High-frequency electrical stimulation is needed for the release of co-transmission neurotransmitters that are primarily stored in large dense-core vesicles (LDCVs)^25–27^. Additionally, adrenergic receptors have been found on the membrane surface of MG 63 cells^15,28,29^. Therefore, the authors concluded that microelectrode stimulation of sympathetic nerve fibers at a low frequency in our study would induce the release of classical neurotransmitters such as epinephrine and norepinephrine, and would consequently influence the formation of new bone in the implanted beagles.

### *In vivo* osseointegration investigation

**Figure 2:**
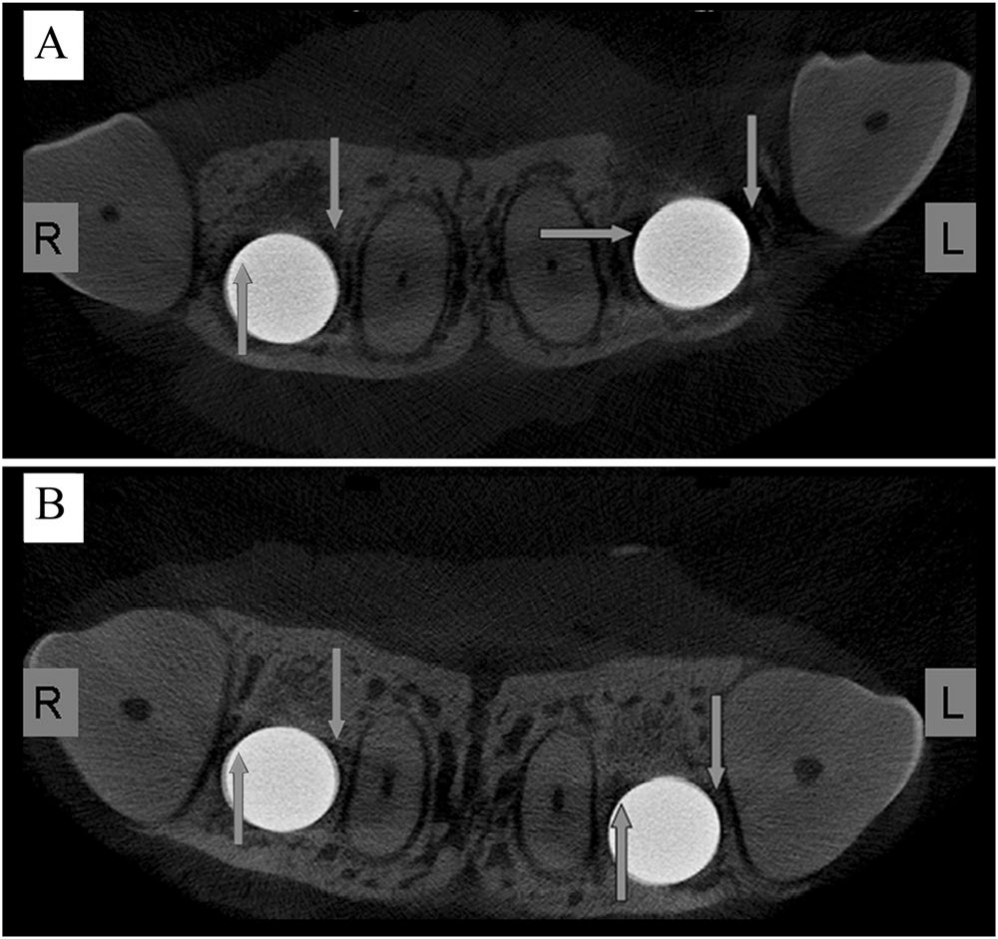
Microelectrode stimulation promotes the osseointegration of implants in beagles. Representative micro-CT images for implants of group A (**A**) and group B (**B**) in the horizontal direction. Group B animals were stimulated by microelectrodes daily for 3 weeks, while group A animals received no treatments and served as controls. The white arrows indicate the gaps between the implants and the surrounding bone tissue.

**Figure 3:**
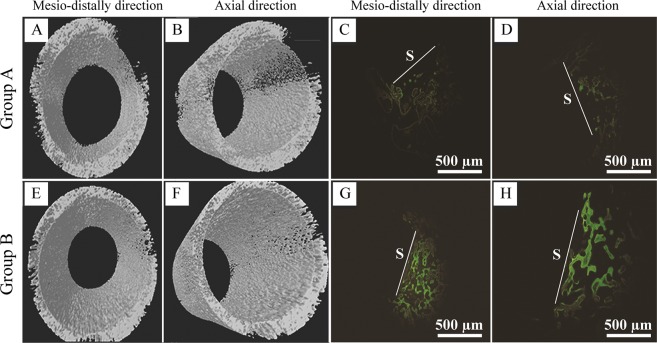
Microelectrodes stimulation facilitated new bone formation. Representative radiographs of micro-CT 3D reconstruction models for surrounding bone of group A (**A**,**B**) and group B (**E**,**F**) implants after implantation for 4 weeks. The new bone formation around implants of group A (**C**,**D**) and group B (**G**,**H**) were examined using bone labeling. Calcein (green) and tetracycline hydrochloride (yellow) were injected on weeks 2 and 4, respectively. White lines indicate the interface between the samples (S) and the new bone. Scale bars, 500 µm.

As detected by micro-CT images in the horizontal direction (Fig. 2), the cortical bone surrounding the implants was essentially healed in the microelectrodes-treated beagles (group B) after 4 weeks of implantation, while obvious gaps were found between the cervical implants and the surrounding bone tissues in the control animals (group A). In addition, three-dimensional (3D) images of the beagle maxilla samples were generated to evaluate the extent of bone formation in tooth defects using the CTAN software package (Fig. 3). When compared to group A (Fig. 3A,B), group B beagles showed increased amounts of new bone surrounding the implants (Fig. 3E,F). Consistently, fluorochrome labeling of calcein (green) that was injected on week 2 showed that group B animals (Fig. 3G,H) exhibited a greater degree of bone regeneration than group A animals (Fig. 3C,D). Although there was an increased yellow signal from tetracycline labeling that was observed at week 4 in group A animals compared to group B, the new bone mass in group B beagles was greater than that of group A. These results demonstrated that microelectrode stimulation of the sympathetic nerve fibers could facilitate the formation of new bone in implanted beagles.

**Figure 4:**
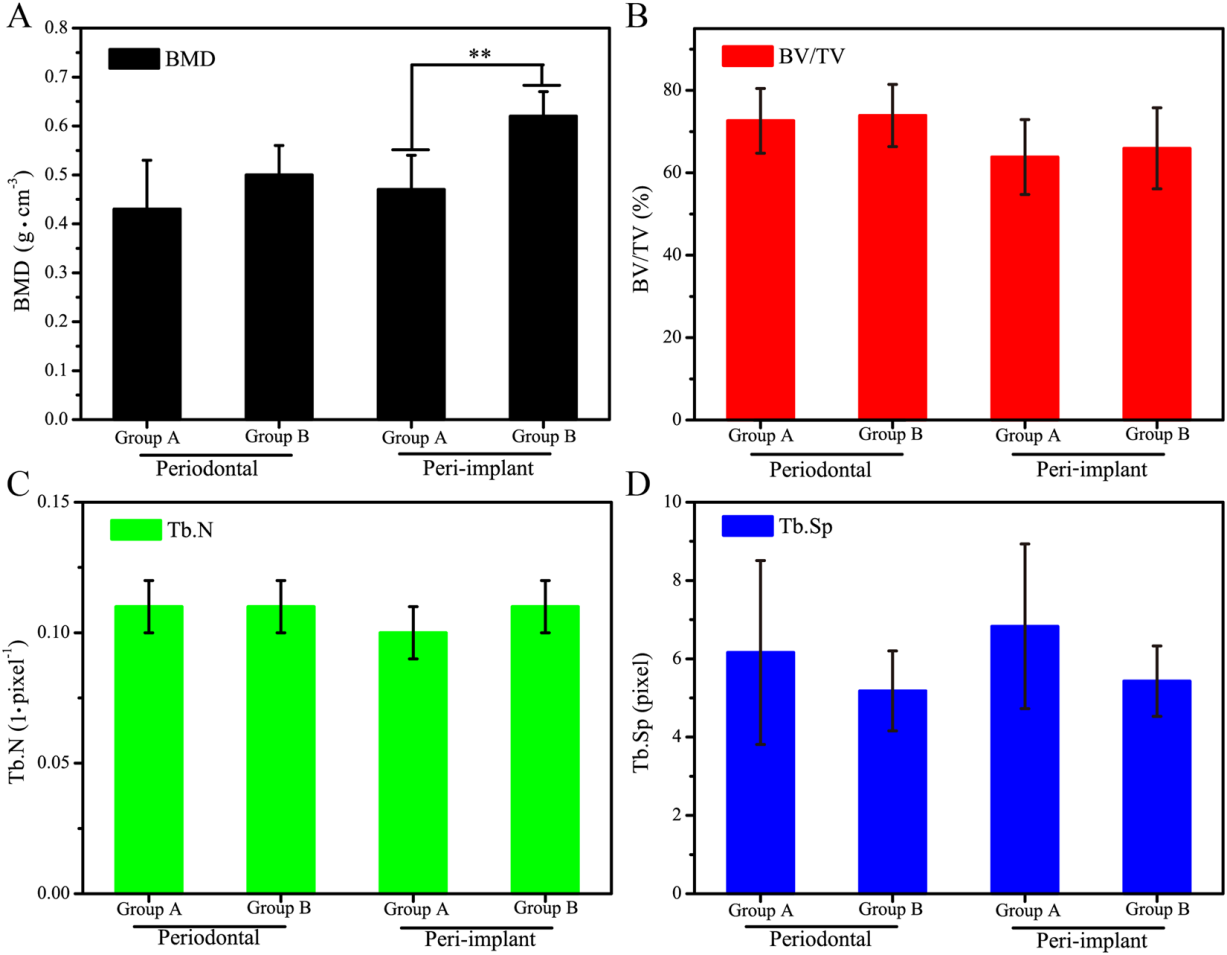
Comparisons of the indicated parameters of periodontal and peri-implant bone between group (**A**) and group (**B**) animals. All measurements were obtained from a micro-CT after implantation for 4 weeks. BMD represents bone mineral density (**A**); BV/TV represents percent bone volume (**B**); Tb.N represents trabecular number (**C**); Tb.Sp represents trabecular spacing (**D**). n = 7, **represents p < 0.01.

Indices such as bone mineral density (BMD), percent bone volume (BV/TV), trabecular number (Tb.N), and trabecular spacing (Tb.Sp) from the 3D micro-CT data were studied to evaluate bone remodeling (Fig. 4). The authors measured these four parameters for the periodontal bone in group A and group B samples, and found no significant differences between the two groups (Fig. 4). This result not only showed that the periodontal bones of the group B animals were nearly the same as those of group A, but also suggested that the microelectrode stimulation of the sympathetic nerve fibers did not affect the existing bone in beagles. Second, the BMD, BV/TV, Tb.N, and Tb.Sp of the peri-implant bone in all beagle samples were measured using bone remodeling (Fig. 4). As shown in Fig. 4a, the BMD of the peri-implant bone for the group B samples (0.62 ± 0.05 g·cm^−3^) was dramatically higher than that observed in group A samples (0.47 ± 0.07 g·cm^−3^) after implantation for 4 weeks (p < 0.01), indicating that the electrical stimulation of sympathetic nerves could increase the mineralization density of the peri-implant bone. The BMD test is generally used to measure the amount of calcium in bone tissue, therefore, the authors concluded that more calcium was deposited in the peri-implant bone of group B compared to group A. Because low bone density is a disadvantageous for the osseointegration of implants, our safe approach of microelectrode stimulation to sympathetic nerve fibers represents a valuable technique for improving the achievement ratio of dental implantations^30,31^. The values of BV/TV, Tb.N, and Tb.Sp of the group B beagles were similar to those of group A (Fig. 4), demonstrating that the microstructure of the new cancellous bone in the implanted beagles (with or without electrical stimulation of sympathetic nerve fibers) was the same. These results also suggest that the electrical stimulation of the sympathetic nerve fibers affects the deposition of bone minerals rather than bone resorption, as was discovered in a previous study that used sympathetic nerve blockers of guanethidine^32^.

It is worth mentioning that both current electric stimulation and pulsed electromagnetic field (PEMF) have been applied to improve the osseointegration process of dental implants^33–36^. However, PEMF stimulation was shown to exhibit no effect on the promoting of the bone-healing process around commercially pure dental implants. For current electric stimulation, a positive result is reported when the implantation time is 2 weeks, but there was no statistically significant difference in new bone formation between the stimulation group and the control group when the healing time was more than 3 weeks. These results suggest that electric stimulation on implanted dental implants is not effective during late-phase implant osseointegration. Fortunately, the authors demonstrate in the present study that electrical stimulation of the sympathetic nerve fibers accelerates the formation of new bone and the osseointegration of implants in beagles after implantation for 4 weeks. This indicates that the up-regulated bioactivity of osteoblasts is evoked by the stimulated nerves during electrical stimulation, but not by the electrical stimulation itself. The primary sympathetic neurons and primary osteoblasts were then isolated from the calvariae and superior cervical ganglia of newly born SD rats, respectively. (Fig. S3). The co-culturing analysis suggested that the cell viability of osteoblasts after 10 days of culture was markedly enhanced when treated with sympathetic neurons compared to the pure osteoblast control (Fig. S4). Moreover, direct material communication between the sympathetic neurons and osteoblasts was found by FRAP microscopy (Fig. S5). These results may provide a preliminary interpretation of why microelectrode stimulation of the sympathetic nerves could promotes new bone formation *in vivo* in implanted beagles, although much effort should made to determine the mechanisms in future studies.

## Conclusions

In the present study, the authors developed a simple and safe physical method using microelectrode stimulation of the sympathetic nerves and confirmed its potential use in promoting implant-bone osseointegration in an animal model. Specifically, the heart rate (HR) and oxygen saturation (SpO_2_) of beagles with implants remained normal during the microelectrode stimulation of the sympathetic nerves of the infraorbital nerve. Significantly consistent curves of electric potential change versus time (s) were detected bilaterally in the infraorbital nerves, although electrical stimulation on the body surface projection of the superior cervical sympathetic ganglion was performed only on the right side. Compared to the control animals with no treatment, there was a markedly increase in new bone surrounding the implants in the microelectrode stimulated beagles after 4 weeks of implantation. Furthermore, the bone remodeling observed in the 3D micro-CT data showed that electrical stimulation of the sympathetic nerve could increase the mineralization density of newly formed bone in the implanted beagles. Additionally, an *in vitro* study on primary rat cell culture found that sympathetic neurons could promote the proliferation of osteoblasts via direct material communication, which may explain the positive impact of sympathetic nerve stimulation on the formation of new bone. These results demonstrate that our method of microelectrode stimulation of sympathetic nerves can promote the formation of new bone and the osseointegration of implants, which has the potential to reduce the healing time of dental implantation in clinical cases.

## Supplementary information

Supporting information
