# Supplementary material for: Nerve electrical stimulation enhances osseointegration of implants in the beagle
Source: Sci Rep. 2019 Mar 20;9:4916. doi: 10.1038/s41598-019-41471-z (PMC6427028; doi:10.1038/s41598-019-41471-z)
Supplement: Supplementary file 1 — Supporting information [file 41598_2019_41471_MOESM1_ESM.docx]

**Supporting Information for**

**Nerve electrical stimulation enhances osseointegration of implants in the beagle**

Ping Zhou^1, 2^, Fei He^1^, Bin Liu^1^* & Shicheng Wei^2, 3^*

^1^ School of Stomatology, Lanzhou University, Lanzhou, Gansu, PR China

^2^ Center for Biomedical Materials and Tissue Engineering, Academy for Advanced Interdisciplinary Studies, Peking University, Beijing, PR China

^3^ Central Laboratory, Peking University School and Hospital of Stomatology, National Engineering Laboratory for Digital and Material Technology of Stomatology, Beijing Key Laboratory of Digital Stomatology, Beijing, PR China

*Address correspondence to Prof. Shicheng Wei (e-mail: [sc-wei@pku.edu.cn](mailto:sc-wei@pku.edu.cn)) or to Prof. Bin Liu (liubkq@lzu.edu.cn)

**Supporting methods**

**Preparation of rat calvarial osteoblasts**

Using a previously reported enzymatic dissociation method, the osteoblasts were obtained from the calvariae of newborn SD rats ^1,2^. Fifteen newborn SD rats (week one) were, respectively, sacrificed by an intravenous injection of 2 ml 3 % pentobarbital. After sterilized in 75 % ethyl alcohol for 5 min, the calvariae of these sacrificed rats were removed from soft tissues in a biosafety cabinet and cut into square slices with a diameter of about 1 mm. Rat calvariae were serially digested with 0.1 % collagenase (Gibco, USA) and 0.25 % trypsin/EDTA (Amresco, USA) at 37 °C. After centrifugation at 1000 rpm for 5 min, cells were resuspended in Dulbecco’s modified Eagle medium (DMEM) (Gibco, USA) containing 10 % fetal bovine serum (FBS; Hyclone, USA), 100 U·mL^-1^ penicillin (Amresco, USA), and 0.1 mg·mL^-1^ streptomycin (Amresco, USA) in 6-well cell culture plates (Corning, USA). Cells were grown under standard cell culture conditions (37 ^o^C, 100% humidity, 95 % air and 5 % CO2). The medium was changed with fresh after culturing for 24 h, aiming to remove nonadherent cells. Cells were fed each two days and passaged into new plates when grown to about 90 % confluence with the treatment of 0.25% trypsin/EDTA.

**Preparation of rat sympathetic neurons**

Isolated sympathetic neurons were acquired from the superior cervical ganglia (SCG) of newborn SD rats as described previously^3,4^. Newborn SD rats were sacrificed and sterilized as mentioned above. As shown in Figure S3, using anatomical marks such as carotid bifurcation, thyroid cartilage and carotid artery, the superior cervical ganglia (SCG) were dissected under a stereo microscope (SZ51, Olypus, Japan) in an bioclean environment, and placed in Hanks' balanced salt solution with 10 mM HEPES (H/H; pH=7.4) at 0 ^o^C. Then, after surrounding fat and connective tissue were removed, each ganglion was cut into small pieces with a sterilized scissor. Ganglion pieces were incubated in 0.25% trypsin/EDTA (Amresco, USA) in a tube (Corning, USA) at 37 ^o^C for 30 min. Same volume medium containing 10 % FBS was added into the tube to neutralize the trypsin. After centrifugation at 1000 rpm for 4 min, cells were resuspended in DMEM (Gibco, USA) containing 10 % FBS (Hyclone, USA), 100 U·mL^-1^ penicillin (Amresco, USA), 0.1 mg·mL^-1^ streptomycin (Amresco, USA) and 50 ng·ml^-1^ recombinant human beta nerve growth factor (β-NGF; Peprotech, USA) in 24-well cell culture plates (Corning, USA). Cells were also grown under standard cell culture conditions (37 ^o^C, 100% humidity, 95 % air and 5 % CO_2_).The medium was changed with medium supplemented with 1 mM 5-fluorouracil (Sigma-aldrich, USA) after culturing for 24 h.

**Cell culture**

Osteoblasts were digested as single cells by 0.25 % trypsin/EDTA (Amresco, USA). After cell counting, osteoblasts were seeded at 1×10^4^ cells·ml^-1^ in culture medium of sympathetic neurons into 24-well cell culture plates with or without attached sympathetic neurons. The medium was changed with fresh in the next day. Cells were then fed each three days. The viability of cells after culturing for various times (3 days, 7 days, and 10 days) were measured using MTT method as previously reported^5^. Briefly, A 100 µL 5 mg·ml^-1^ MTT (Sigma-Aldrich, USA) solution (PBS buffer, pH=7.4) was added into each well of 24-well cell culture plates, and incubated at 37°C in a 5% CO_2_ buffered and humidified incubator (SANYO, Japan) for 4 h. Then, for each well, 100 µL sodium dodecyl sulfate (SDS; Amresco, USA) solution was applied to dissolve the formed blue crystals of formazan. The absorbance value (OD value) was measured at wavelength 560 nm and 620 nm with a microplate reader (BIO-RAD 680, Bio-Rad, CA). Moreover, we quantitative measured the ALP activity of cells after culturing for various times (3 days, 7 days, and 10 days). The culture medium in each well of 24-well cell culture plates were transferred into a tube respectively. A 150 µL solution containing 25 mmol·L^-1^ diethanolamine, 1 mmol·L^-1^ magnesium chloride and 6.7 mmol·L^-1^ p-nitrophenyl phosphate (PNPP) was injected into each tube. After incubation at 37 ^o^C for half an hour in the dark, each 100 µL 0.1 mmol·L^-1^ sodium hydroxide solution was added into a tube to end the reaction. Then, these tubes were centrifuged at 1000 rpm for 5 min. The absorbance value (OD value) of supernatants was measured in a 96-well cell culture plate at wavelength 405 nm with a microplate reader. Both for MTT and PNPP analysis, three specimens were tested for each incubation period, and each test was performed in triplicate.

**Fluorescence recovery after photo-bleaching (FRAP) microscopy**

The osteoblasts and sympathetic neurons were co-cultured in glass-bottom culture dishes with a diameter of 35 mm for 7 days as mentioned previously. The cells were rinsed with Hanks' balanced salt solution twice and then incubated with 10 μg·ml^-1^ 6-Carboxyfluorescein Diacetate solution at 37°C in a 5% CO_2_-buffered and humidified incubator for 20 min. After being cleaned with Hanks' balanced salt solution several times, a culture medium of sympathetic neurons was added into each of the dishes. The FRAP measurement was performed using a Leica TCS-SP live confocal laser scanning microscope. A single osteoblast was bleached with a 488 nm laser beam at 100% laser power. Fluorescence recovery of the cells was recorded every 5 s for a total of 45 s. The fluorescence recovery at every time point was measured as previously described^6,7^.

**Supporting results**

**Sympathetic neurons promote osteoblast proliferation**

To determine the mechanism underlying the effect by which micro-electrode stimulation of sympathetic nerves promotes new bone formation, we isolated primary sympathetic neurons and primary osteoblasts from the calvariae and superior cervical ganglia of newly born SD rats, respectively. Sympathetic neurons were co-cultured with osteoblasts for different periods (24 h, 48 h, 72 h), and their viability and ALP activity were investigated (Fig. S4). After culturing for 48 h, many synapses in the sympathetic neurons connected with osteoblasts, and physical contact was found for sympathetic neurons and osteoblasts when cultured for 72 h (Fig. S4A). Moreover, cell proliferation was observed for osteoblasts but not for sympathetic neurons, because sympathetic neurons are terminally differentiated somatic cells (Fig. S4A). A co-culture analysis determined that after 10 days of culture, the cell viability of osteoblasts was markedly enhanced with the treatment of sympathetic neurons (Fig. S4B) but that the ALP activity values were almost the same throughout the study (Fig. S4C). These results demonstrate that the secretion from sympathetic neurons could significantly accelerate the proliferation of osteoblasts.

Fluorescence recovery after photo-bleaching (FRAP) analysis was conducted for co-cultured sympathetic neurons and osteoblasts, and the fluorescence of bleached osteoblasts and selected surrounding regions was measured at 5-s intervals for 45 s using a laser confocal microscope (Fig. S5). The fluorescence of the bleached region in osteoblasts was increased with an additional measuring period of 35 s. A decrease in fluorescence was found in the surrounding osteoblasts immediately after fluorescence bleaching was performed. Although no significant change was measured for the fluorescence of sympathetic neurons after 5 s in recovery, sympathetic neurons presented the largest decrease in fluorescence when the recovery time increased to 15 s. In this study, sympathetic neurons contact osteoblasts through synapses, which means that a longer response time is needed compared to directly contacted osteoblasts. FRAP microscopy showed that direct material communication existed between sympathetic neurons and osteoblasts, which could be why micro-electrode stimulation on sympathetic nerves promotes the proliferation of osteoblasts *in vitro* and the formation of new bone *in vivo*.

**Supporting figures**

**
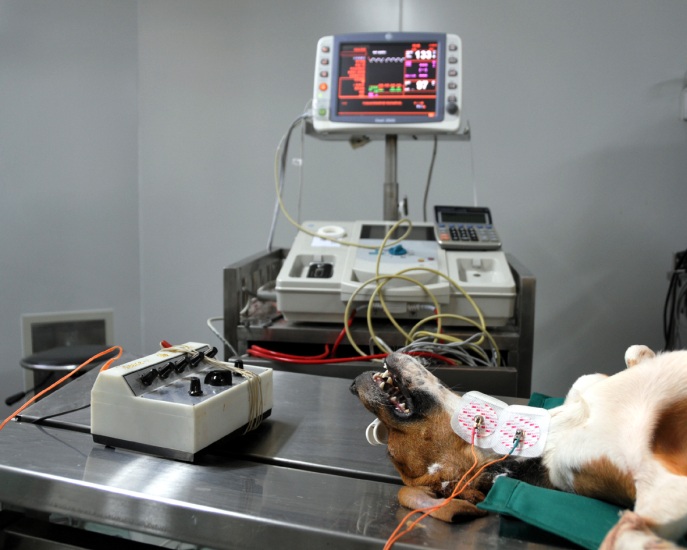
**

**Figure S1.** Photo for electrical stimulations on sympathetic nerve of beagles with implants.





**Figure S2.** The heart rate (HR) and oxygen saturation (SpO_2_) of beagles after stimulation by micro-electrodes for various times (0 min, 1 min and 3 min).


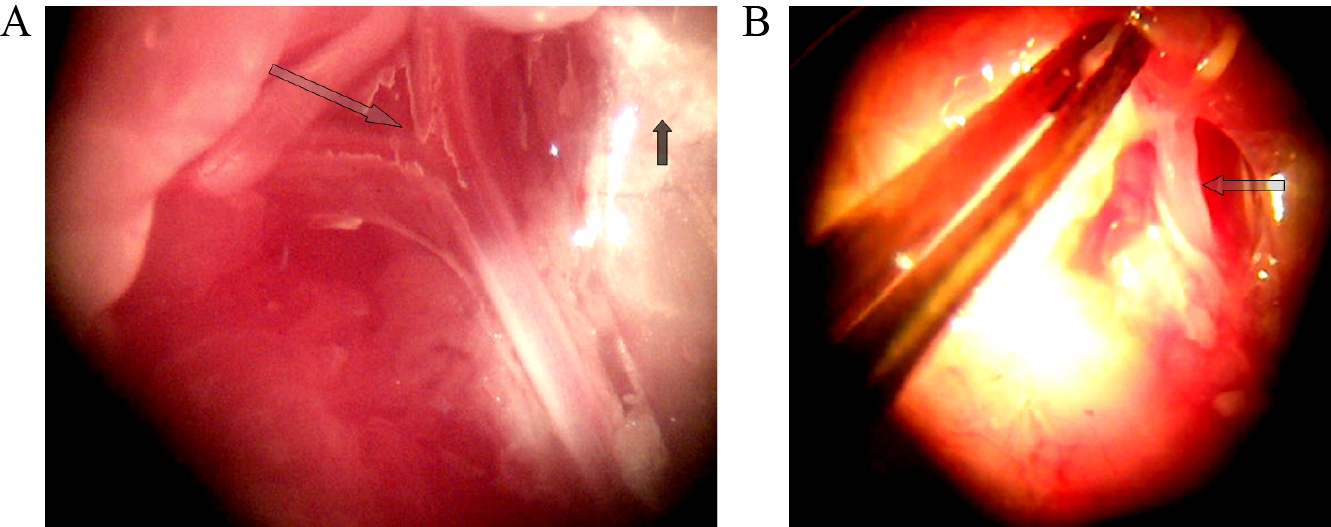


**Figure S3.** Images of dissect process for superior cervical ganglia (SCG) under a stereo microscope. (A) Carotid bifurcation and thyroid cartilage were shown by a transparent arrow and a black arrow respectively. (B) Image of the cervical sympathetic, back of carotid artery, was shown by a transparent arrow.


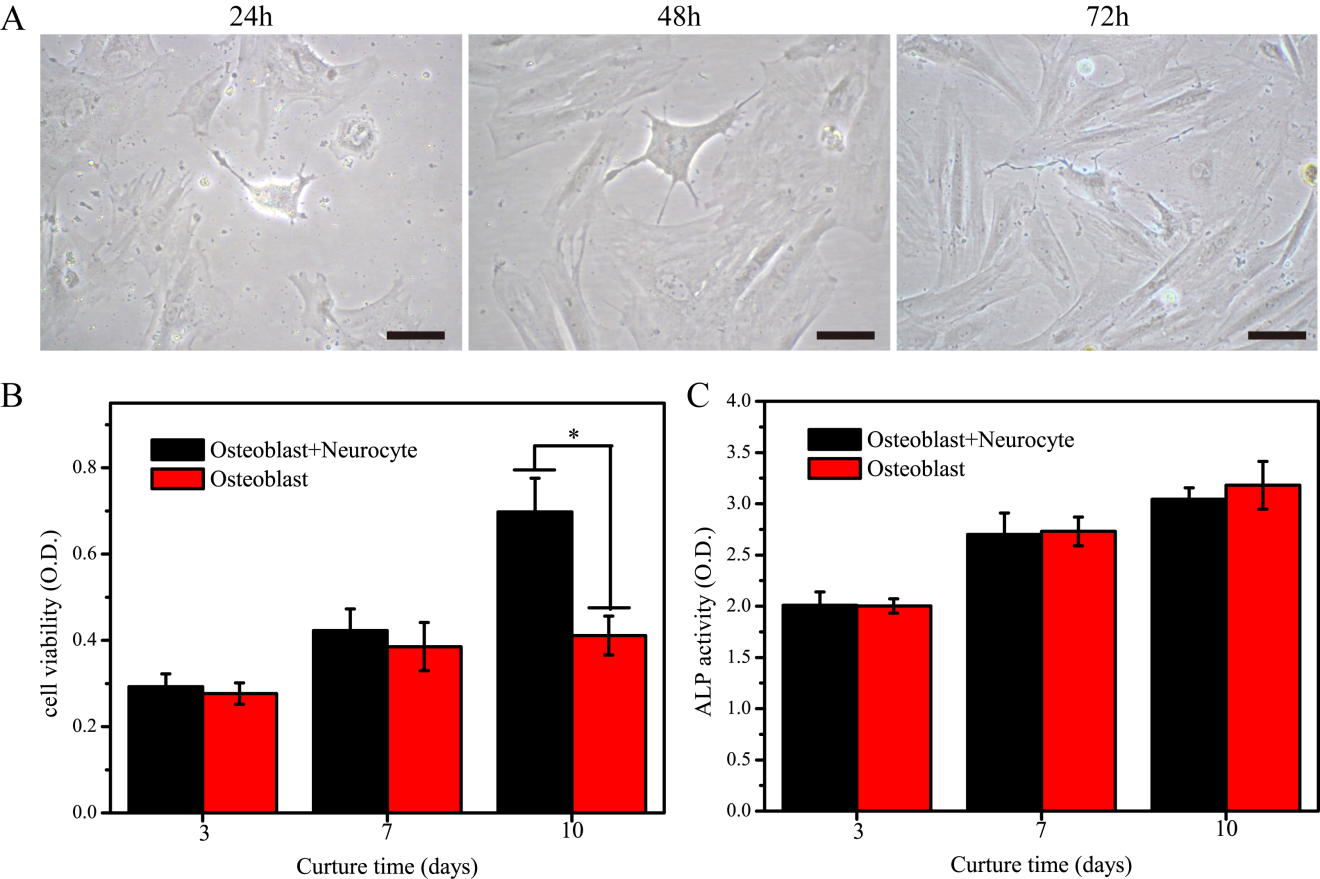


**Figure S4.** The proliferation of osteoblasts was apparently promoted when co-cultured with sympathetic neurons. (**A**) Representative morphology of osteoblasts and sympathetic neurons after co-culturing for different periods (24 h, 48 h, 72 h). Scale bars, 100 µm. (**B**) The viability of the indicated cells after culturing for various days (3 days, 7 days, 10 days) was measured using the MTT method. (**C**) The ALP activity of those cells samples was detected by the quantitative PNPP analysis. *Represents p < 0.05 (n = 3).


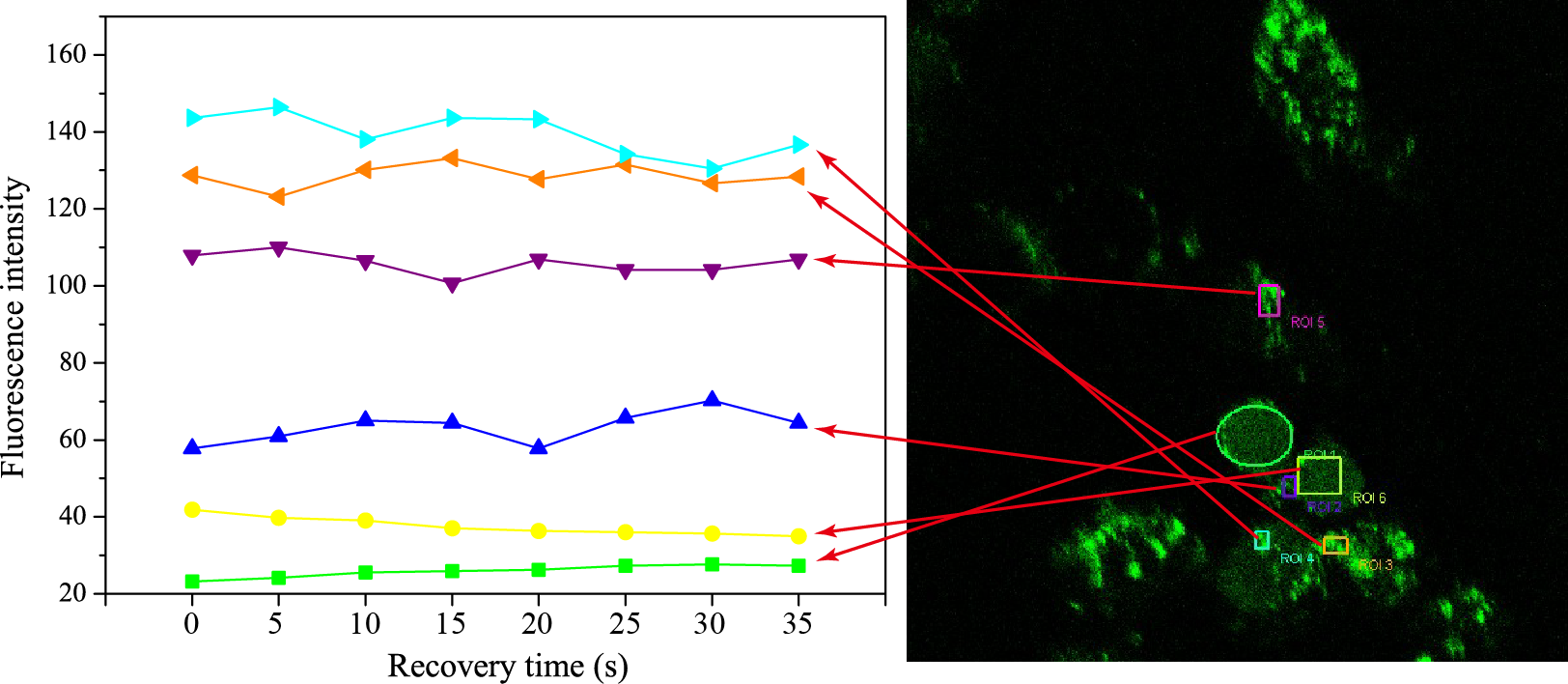


**Figure S5.** Fluorescence recovery after photo-bleaching (FRAP) analysis. A single osteoblast, labeled with a circle, was bleached by 488 nm laser beam with 100% laser power. The fluorescence of bleached cells and other five indicated region that marked with various colors boxes was measured at 5 s interval for 35 s using a laser confocal microscope.

**References**

1. Onodera, S. *et al.* Macrophage Migration Inhibitory Factor Up-regulates Matrix Metalloproteinase-9 and -13 in Rat Osteoblasts RELEVANCE TO INTRACELLULAR SIGNALING PATHWAYS. *J. Biol. Chem.* **277**, 7865 (2002).

2. Wong, G. & Cohn, D. V. Separation of parathyroid hormone and calcitonin-sensitive cells from non-responsive bone cells. *Nature* **252**, 713-715 (1974).

3. Blennerhassett, M. G., Tomioka, M. & Bienenstock, J. Formation of contacts between mast cells and sympathetic neurons in vitro. *Cell Tissue Res.* **265**, 121 (1991).

4. Zareen, N. & Greene, L. A. Protocol for culturing sympathetic neurons from rat superior cervical ganglia (SCG). *Jove*, 219-222 (2009).

5. Stoev, S., Denev, S., Dutton, M. & Nkosi, B. Cytotoxic Effect of Some Mycotoxins and their Combinations on Human Peripheral Blood Mononuclear Cells as Measured by the MTT Assay. *Open Toxinology Journal* **2** (2009).

6. Lemcke, H., Nittel, M. L., Weiss, D. G. & Kuznetsov, S. A. Neuronal differentiation requires a biphasic modulation of gap junctional intercellular communication caused by dynamic changes of connexin43 expression. *Eur. J. Neurosci.* **38**, 2218-2228, doi:10.1111/ejn.12219 (2013).

7. Lemcke, H. *et al.* Applying 3D-FRAP microscopy to analyse gap junction-dependent shuttling of small antisense RNAs between cardiomyocytes. *J. Mol. Cell. Cardiol.* **98**, 117-127 (2016).
